# Dr. Edward E. Koehler

**Published:** 1915-02

**Authors:** 


**Dr. Edward E. Koehler,** Niagara University, 1894, died at his home in Buffalo, of tuberculosis, January 3, 1915, aged 44.

